# 26. A case of an autoinflammatory condition with cerebrovascular infarctions

**DOI:** 10.1093/rap/rky033.017

**Published:** 2018-09-20

**Authors:** Emma J Davies, Harsha Gunawardena

**Affiliations:** Rheumatology, North Bristol NHS Trust, Bristol, UNITED KINGDOM

**Introduction:** We report the case of a 43 year old who presented with shortness of breath and pleuritic chest pain. She rapidly developed multi-organ involvement with multiple stoke like episodes, acute kidney injury, transaminitis and disseminated intravascular coagulopathy. This was associated with quotidian fevers and a significant acute phase response. Following extensive investigation her clinical features were felt to be most consistent with an undifferentiated autoinflammatory disorder. She had limited a response to multiple therapeutic interventions including empirical steroids but had a rapid and sustained response to IL-1 blockade with anakinra.

**Case description:** A 43 year old female with a background of chronic urticaria and episodes of recurrent pleurisy treated in the community as presumed infection was admitted via the emergency department with chest pain and dysponea. She was initially diagnosed with right upper zone community acquired pneumonia and commenced on antibiotics. Ten days following admission she developed an expressive dysphasia, confusion and a quadrantinopia. Areas of presumed acute infarction were reported in the left parietal and occipital lobes on a CT scan of her head. Subsequent MRI demonstrated areas of high T2 signal in the left middle cerebral artery territory without significant vessel disease. During her admission, she was noted to have recurrent periodic fevers and had a significant inflammatory response (CRP >180, ferritin >3000, anaemia, transaminitis). Six weeks into her hospital admission she deteriorated and developed type 2 respiratory failure. She was transferred to the intensive care unit for intubation, ventilation and inotropic support. She was found to have a right sided collapse on chest radiograph and transoesophageal echocardiogram was consistent with secondary pulmonary hypertension. She developed an acute kidney injury, requiring haemofiltration, and disseminated intravascular coagulaopathy. In view of her clinical deterioration empirical treatment with intravenous methylprednisolone and plasma exchange were commenced. Her admission was further complicated by a complex left hydropneumothorax with a probable bronchopulmonary fistula, most likely due to persistent pleural inflammation. An extensive infection screen was negative. A full autoimmune screen was also normal/negative. Repeat MRI head showed further high T2 signal involvement without diffusion restriction involving the basal ganglia, thalami, corpus callosum, brainstem, cerebellar hemispheres and subcortical white matter of both cerebral hemispheres. It was felt that the absence of restricted diffusion involving the new lesions made infection and infarction less likely and the symmetry of the changes was suggestive of a metabolic or inflammatory aetiology. Due to the severe multi-organ nature of her illness she underwent numerous biopsies including renal, liver, muscle, skin and bone marrow. The liver, renal and muscle biopsies did not aid diagnostically, all showing necrosis without signs of vasculitis. The skin and bone marrow trephine were not diagnostic but were supportive of an autoinflammatory condition such as adult onset Still’s disease (AOSD). The first skin biopsy showed very subtle changes but the third showed widespread epidermal dyskeratosis with modest inflammatory infiltrate. The bone marrow aspirate showed myeloid lineage with maturation and an increase in megakaryocytes with possible haemophagocytosis. Ten weeks following admission with no response to multiple courses of antibiotics, limited response to empirical steroids and plasma exchange it was concluded that the clinical presentation and investigations were most consistent with a diagnosis of an autoinflammatory disorder (possibly within the AOSD spectrum). She was commenced on anakinra and had a rapid and sustained improvement. She has remained stable throughout follow up on anakinra and low dose prednsiolone, apart from secondary epilepsy due previous cerebral damage managed with levertiracetam and lamotrigine.

**Discussion:** We report a case of subacute onset of a multisystem disorder leading to multiorgan failure. This patient had been on the intensive care unit for eight weeks prior to the request for a rheumatology review. Given the history and investigation results along with the numerous negative results we felt the most likely diagnosis was an autoinflammatory condition such as AOSD or deficiency of adenosine deaminase 2 (DADA2). Adult onset Still’s disease is a rare but well recognised condition. The commonest features are: fevers, rash and arthralgia with an acute phase response. Disseminated intravascular coagulopathy, pleurisy, deranged liver function tests and renal involvement are all rare but recognised features. An alternative potential diagnosis, for which she has been tested and the results are awaited, is DADA2. This is a rare autosomal recessive autoinflammatory condition. Common features include: intermittent recurrent fevers, a livedo reticularis rash, vasculopathy and a high risk for early onset strokes. Given the likely diagnosis and her response to plasma exchange and pulsed IV methylprednisolone, with subsequent deterioration we felt that IL-1 blockade with anakinra was the most appropriate treatment option. The patient recovered and has remained well on anakinra.

**Key Learning Points:** Autoinflammatory conditions are usually a clinical diagnosis (at least in the acute phase) with no specific tests. Sometimes we have to work on a best fit theory based on the clinical information. Plasma exchange is a relatively safe treatment option in patients who are very unwell, especially where infection remains a possibility. Strokes in young people have a large number of causes - including some less well known e.g. DADA2. Complex neurological and rheumatological patients often overlap.

**Disclosure: E.J. Davies:** None. **H. Gunawardena:** None.

